# Cyclosporine Inhibits a Direct Interaction between Cyclophilins and Hepatitis C NS5A

**DOI:** 10.1371/journal.pone.0009815

**Published:** 2010-03-23

**Authors:** Fiona Fernandes, Israr-ul H. Ansari, Rob Striker

**Affiliations:** 1 Department of Medicine, University of Wisconsin-Madison, Madison, Wisconsin, United States of America; 2 W. S. Middleton Memorial Veterans Administration Hospital, Madison, Wisconsin, United States of America; Karolinska Institutet, Sweden

## Abstract

**Background:**

Hepatitis C Virus (HCV) infection is a leading indication for liver transplantation. HCV infection reoccurs almost universally post transplant, decreasing both graft longevity and patient survival. The immunosuppressant, cyclosporine A (CsA) has potent anti-HCV activity towards both HCV replicons and the genotype 2a cell culture infectious virus. Previously, we isolated mutations in the 1bN replicon with less sensitivity to CsA that mapped to both NS5A and NS5B regions of the virus. Mutations in NS5A alone conferred decreased CsA susceptibility regardless of NS5B mutations.

**Methodology/Principal Findings:**

We examined the mechanisms by which NS5A mutations contribute to CsA resistance and if they are strain dependent. Using in vitro mutagenesis, the amino acid position 321 mutation of NS5A was restored to the wild-type tyrosine residue conferring partial CsA susceptibility on the mutant replicon. The 321 mutation also alters CsA susceptibility of the JFH cell culture virus. Additionally, we demonstrated a novel CsA-sensitive interaction between NS5A and both cyclophilin A and B. Both the mutant NS5A and wild type NS5A bind cyclophilin in vitro. The NS5A: cyclophilin interaction requires both the NS5A region identified by the resistance mutants and cyclophilin catalytic residues. In cell culture, NS5A from CsA resistant mutant has an enhanced interaction with cyclophilin B. Additionally; NS5B facilitates a stronger binding of mutant NS5A to endogenous cyclophilin B than wild-type in cell culture.

**Conclusions/Significance:**

Collectively, this data suggests direct interactions between cyclophilins and NS5A are critical to understand for optimal use of cyclophilin inhibitors in anti-HCV therapy.

## Introduction

HCV infects 200 million people worldwide. Current treatment options for HCV include pegylated interferon α and ribavirin. However this treatment is not always effective or tolerated well [1]. Thus, new therapies are urgently needed. Frequently, patients do not have symptoms from HCV infection until their liver is severely damaged. Liver transplantation becomes the only practical means of restoring health and requires long-term immunosuppression to prevent graft rejection. Almost every transplant patient receives one of two different calcineurin inhibitors, either tacrolimus or CsA. CsA and its nonimmunosuppressive analogs, DEBIO-25 and NIM811, show in vitro antiviral activity against the HCV 1a, 1b, and 2a experimental replicons that is related to their ability to inhibit a class of cellular Prolyl-peptidyl isomerases called cyclophilins (Cyp) [2], [3], [4]. Tacrolimus lacks this activity [4], [5]. However, for transplant patients infected with HCV, it remains unclear if CsA or tacrolimus is the calcineurin inhibitor of choice.

It was initially proposed that in vitro inhibition of HCV by CsA results from disruption of the interaction between the HCV polymerase, NS5B, and Cyclophilin B (CypB) [6]. CypB enhances binding of NS5B to RNA [6], [7], [8]. A previous study showed CsA addition to HeLa cell cultures results in relocalization and secretion of CypB [9]. These findings were further corroborated by recent clinical data from HCV and HIV co-infected patients. The treatment of these co-infected patients with the CsA analog Debio-025 results in a decrease of CypB levels in peripheral blood mononuclear cells [10]. This decrease in CypB levels coincides with a decrease in hepatitis C viral load. In contrast, Debio-025 treatment does not lead to decreases in Cyclophilin A (CypA) levels [10]. While results are conflicting, siRNA data is most consistent with CypA rather than CypB playing major role in HCV subgenomic RNA replication [4], [6], [11], [12], [13]. Whether all HCV replicons have the same cyclophilin requirement is unclear [11]. Both CypA and CypB interact with the HCV NS5B polymerase [13], [14].

We previously isolated HCV 1bN replicon sequences more resistant to CsA treatment [15]. Resistance mapped to two regions of the HCV genome, NS5A and NS5B. All CsA resistant replicons contain a set of similar, but not identical NS5A mutations, with essentially the same NS5B mutations. Additionally, a mutant replicon CsA-1s containing NS5A mutations alone confers as much resistance as CsA-1s replicons containing mutations in both NS5A and NS5B. Whether NS5A mutants are directly altering the susceptibility of HCV replication to CsA, or indirectly modulating the NS5B: cyclophilin interaction is unclear.

In this study we present data supporting a direct role of NS5A in CsA resistance. The NS5A mutations from the 1bN replicon also confer CsA resistance for the canonical Con 1b replicon. The tyrosine residue at position 321 of NS5A is a particularly critical determinant of CsA resistance. This residue is within the ‘277-334’ NS5B binding region of NS5A [16], but does not influence the NS5A-NS5B interaction. We also demonstrate NS5A can bind either CypB or CypA. A mutant NS5A is described that is more resistant to the cellular effects of CsA inhibition despite a similar biochemical binding to cyclophilins as wild-type NS5A. The interaction between CypA and NS5A depends upon both catalytic residues of CypA and domain 2/3 regions of NS5A where the cell culture derived mutants map. In cell culture, the mutant shows enhanced binding to CypB compared to wild-type NS5A. Additionally, NS5B promotes stronger binding of the mutant NS5A to CypB than wild- type NS5A. NS5A binding to cyclophilins may thus be a sensitive determinant of the overall susceptibility of HCV to cyclophilin inhibitors.

## Materials and Methods

### Ethics Statement

The funders had no role in study design, data collection and analysis, decision to publish, or preparation of the manuscript.

### Cells and Plasmids

EN5.3 and Huh7.5 cells were used for all studies. Huh7.5 cells and the Con1 HCV replicon were generous gifts from Dr. Charles Rice. For these studies, the neomycin resistance gene was replaced with a renilla luciferase-neo fusion gene amplified from a replicon kindly provided by Drs. Ikeda and Kato [17]. The pNtat2ANeo/SI plasmid containing the HCV 1bN consensus sequence (GenBank accession no. AF139594) with an adaptive serine to isoleucine change in the NS5A S232I was provided by Dr. Lemon [18]. After transfection with replicon RNA, EN5.3 cells were selected and maintained in growth media containing blasticidin and 0.5–1.0 mg/ml G418 (Geneticin, Invitrogen, La Jolla, CA). Huh7.5 cells were transfected and maintained in complete growth media supplemented with non-essential amino acids and 0.5 mg/ml G418.

### Genetic manipulation and cloning of HCV Replicon

The CsA-3s NS5A (acc. No. EF115302) cloning was performed as previously described [15]. The Chimeric Con 1b replicon was generated as follows. Primers were designed to amplify two separate PCR fragments from the Ntat/SI replicon genome to eliminate the 4 amino acid SSYN insertion. New amplicons were generated by overlap PCR using ultra Pfu polymerase (Stratagene, La Jolla, CA). The fragments were cloned into a TOPO TA vector (Invitrogen, Carlsbad, CA). The TOPO constructs were digested with BlpI and ClaI and cloned into the original Con Luc Neo plasmid vector. A similar cloning procedure was employed to obtain Chimeric 3s. Quick-change mutagenesis was performed using Topo Chimeric 3s amplicon as a template. To generate different Chimeric 3s replicon variants, the BlpI –ClaI fragment was inserted into the Con Luc neo plasmid to generate Chimeric 3s G256E, Chimeric 3s A280V, Chimeric 3s Q303L, Chimeric 3s N321Y, Chimeric 3s G356R and Chimeric 3s A444V replicons.

### RNA transcription and transfection

Replicon DNA was linearized with XbaI (NEB, Ipswich, MA) and transcribed using a T7 Ampliscribe Flash transcription kit (Epicenter Biotechnology, Madison, WI). RNA was transfected into EN5.3 or Huh7.5 cells using the Transit mRNA transfection kit (Mirus, Madison, WI). Three weeks after the establishment of stable replicon cell lines using G418 selection, equal numbers of replicon cells were seeded into 6 well plates with or without CsA. The Secreted Alkaline Phosphatase (SEAP) assay was used to quantitate the Ntat replicon RNA as described previously [19]. To assay the Renilla luciferase activity, Con Luc Neo replicon cells were lysed with 100 µl of Renilla Lysis buffer supplied with the Renilla Luciferase Assay system (Promega, Madison, WI). 10 µl of clarified cell lysate was added to 50 µl of Renilla Luciferase Assay buffer and read in triplicate on a Turner Designs TD20/20 Luminometer (Promega, USA).

Following the establishment of stable replicon cell lines, RNA was isolated using the Trizol method. Total RNA was used for cDNA synthesis using a primer located in N-terminal region of NS5B. PCR was performed using this reverse primer and a forward primer located within NS5A region. PCR product of the expected size was excised from gel, purified and cloned into the TOPO vector. Two clones from each mutant replicon were sequenced with M13 forward and M13 reverse primers. The results were analyzed and specific mutations were confirmed by checking the chromatogram.

### Transient Replication Assay

In vitro transcribed Con 1b RNA along with Chimeric 3s were electroporated into ∼4×10^6^ Huh7.5 cells. Following RNA electroporation, one tenth of the cells were set aside to check the luciferase activity at 4 hrs as an indicator of RNA electroporation efficiency. The rest of the electroporated cells were equally divided and plated into 24 wells so that each electroporation had 3 sets of 8 wells. Four wells of each set were left untreated while wells 5–8 were treated with 1.0 µg/ml of CsA until the assay was performed. The cells were collected at 4 time points (24, 48, 72 and 96 hrs) post RNA electroporation from both CsA treated and untreated wells. Luciferase activity was monitored as above.

To monitor the replication assay in Huh7.5 cells, the cells were pretreated with 1.0 µg/ml CsA for one week. These cells were electroporated with either Con 1b or Chimeric 3s replicon RNA and luciferase activity was monitored at 24, 48, 72 and 96 hrs. The electroporations were performed in triplicate and average luciferase activity for both Con1b and Chimeric 3s is presented.

### Infectious virus recovery and Northern Blot assay

Infectious HCV particles were produced as described before [20], [21]. To observe the effect of CsA on infectious virion production by JFH-wt and JFH-NS5A-Y325N (JFH-NS5A-Y325N corresponds to the Y321N in Table 2A), 10 µg in vitro synthesized RNA was electroporated in normal cells and in cells pre-treated with 1.0 µg/ml CsA for one week. After 5 days, the supernatants were collected, filtered through 0.45 µM filters and passed onto naïve cells plated the day before. After 12 hrs, supernatant were removed and either complete growth media or media containing CsA was added. The plate was further incubated for 72 hrs and Immunofluorescence assay was performed using mAb to NS5A as described before [20].

For northern blot assay, the same amount of RNA from JFH1-wt and a JFH1-Y325N mutant was electroporated into normal Huh7.5 cells and cells already treated with 1.0 µg/ml of cyclosporine for one week. The CsA-treated and electroporated cells were further incubated in presence of CsA while normal cells were grown in complete growth media. After 72 hrs post RNA electroporation, cells were lysed in Trizol (Invitrogen, CA) and total RNA was isolated following manufacturer's protocol. The northern blot was performed using standard molecular biology protocols [22], [23]. Approximately 20 µg of RNA was resolved by glyoxal gel. The RNA was transferred onto nylon membrane and cross-linked using UV. The probe was made with a Random Priming DNA Labeling Kit (Roche, USA) using a DNA template derived from NS5A C-terminal region and purified using Illustra ProbeQuant G-50 Micro Columns (GE Health, USA). The membrane was incubated with the above probe overnight and washed once with low stringency and twice with high stringency wash buffer provided with the NorthernMax kit (Applied Biosystems). Hybridization of radiolabeled probe was visualized using autoradiography and quantified using Storm followed by image quant to obtain respective values. Data was normalized with respect to actin gene hybridization for presentation.

### Mammalian expression constructs

The wt NS5A-His (1bN-SI) and mt NS5A-His (CsA-3s) constructs were made as follows. The NS5A genes were amplified from the Ntat/SI and CsA-3s replicon by RT-PCR. Forward primers were designed with an XhoI site and an in-frame start codon ATG 5′ to the NS5A sequence. The reverse primers encoded a 6XHis tag in frame with the NS5A sequence, a stop codon followed by Pac1 restriction site. The resulting PCR product was cloned into XhoI-PacI digested pIRES/GFP (Green Fluorescent Protein) mammalian expression vector (kindly provided by Dr. Kouacou Konan). The HA-wt NS5A and HA-mt NS5A constructs were made by incorporating the HA tag (MYPYDVPDYA) in the forward primer. The GFP gene in these constructs was removed and the plasmid recircularized with T4 DNA ligase. For the N terminal truncated NS5A constructs lacking domain 1 (D23NS5A), forward primers were designed to encode a XhoI site, Kozak sequence, HA, the ATG start codon, and NS5A sequence starting from amino acids 148 for both the 1bN and the CsA-3s replicon DNA templates. The reverse primers contained an in-frame stop codon and a Pac1 site. The GFP gene in these constructs was also removed and religated with T4 DNA ligase. The Con1b NS5B was generated by using standard PCR approach. The forward primer was designed to contain a FLAG epitope (MDYKDDDDK) and cloned in a CMV-based mammalian expression vector. The CypB mammalian expression vector was generated with forward primers containing XhoI, ATG 5′ to the start of the CypB sequence and reverse primers containing an HA tag, stop codon and a PacI site. The PCR fragment was then cloned into the pIRES/GFP vector.

### Immunoprecipitations

HA-wt NS5A and HA-mutant (mt) NS5A expression constructs were co-transfected with a FLAG-tagged Con 1b NS5B expression construct in 293T cells using transit LT1 reagent (Mirus, Madison, WI). Forty-eight hours after transfection, cells were harvested and immunoprecipitated with HA-specific antibody (Biodesign). Immunoprecipitates were separated using SDS-10% Poly Acrylamide Gel Electropheresis (PAGE), and western blotting was performed. Blots were probed with a monoclonal NS5A-specific antibody (Biodesign) and a monoclonal FLAG antibody against the FLAG tag of NS5B (Sigma). An HRP-linked mouse secondary antibody specific for antibody light chains (Jackson Immunological Research) was used and the signal was developed using Immun-star HRP chemiluminescent substrate (Bio-Rad, Hercules, CA). For NS5A and CypB transfections, wt or mt NS5A-His and HA tagged CypB were immunoprecipitated with His-specific monoclonal antibody (Qiagen) bound to protein G agarose beads, washed, and loaded onto a gel. The blots were probed with a NS5A monoclonal (Biodesign) and a rabbit polyclonal to the HA tag. For the NS5A and NS5B cotransfections, HA-wt NS5A and HA-mt NS5A were cotransfected in 293T cells with FLAG Con1b NS5B. The lysates were immunoprecipitated with HA-specific antibody and probed with NS5A-, FLAG-, and CypB-specific antibodies to check levels of NS5A, NS5B and CypB respectively.

### Cyclophilin binding assay

Recombinant CypB was obtained as previously described [15]. GST-CypA was a generous gift from Dr. Carol Carter. Full-length (HA-wt NS5A or HA-mt NS5A) or N-terminal truncated NS5A constructs (D23NS5A), as well as N terminal FLAG-tagged Con 1b NS5B were in vitro translated in the presence of ^35^S methionine/Cysteine (Expre^35^S^35^S protein labeling mix, NEN Life Sciences, MA) using the TNT Quick coupled Transcription/Translation Systems (Promega, Madison USA) following the manufacturer's protocol. The assay for GST-CypB binding to NS5A or NS5B was performed as previously described [24]. In brief, the radiolabelled proteins HA-wt NS5A and HA-mt NS5A were incubated with approximately 2.0 µg of E.coli-expressed and purified GST-CypB in binding buffer overnight. The bound complex was washed 5 times with PBS containing 0.25% NP-40. The complex was mixed with 2× SDS-PAGE sample buffer, boiled for 5 minutes, and analyzed by SDS-10%PAGE along with protein molecular weight markers (Precision Plus Protein Standard; Bio-Rad). The signal was developed after fluorography. In a similar fashion the radiolabelled NS5A was incubated with purified GST-CypA and binding was assessed. For CsA inhibition in the binding experiment, drug was added to the binding buffer followed by addition of NS5A and GST-CypA.

To further confirm the specificity of CypA NS5A binding, an irrelevant protein, HIS tagged Cyan fluorescent protein (CFP) was expressed in E. coli and purified using Ni^++^ column following manufacturers protocol (Qiagen). Approximately 2.0 µg of CFP and NS5A proteins were incubated with equal amounts of purified GST or GST-CypA proteins and binding was performed as described before [24]. The complexes were resolved using SDS-10% PAGE, and probed with anti-HIS monoclonal antibodies (Qiagen) to detect HIS tagged proteins.

To map the region within NS5A responsible for binding, a series of in-frame 90 amino acids deletion mutants were generated in the HA-wt NS5A expressing plasmid. These constructs were in vitro translated in presence of ^35^S methionine/cysteine and incubated with purified GST-CypA. The binding was performed as above. The signal was developed after fluorography and intensity of individual pull down signal was quantified using image quant following Storm. To further map the binding region within CypA, the two active site residues (R55 and F60) were mutated to Alanine. The ^35^S radiolabelled NS5A was again incubated with either wt GST-CypA or mt GST-CypA (carrying the two active site mutations) and binding was performed as described above.

### Statistical Analyses

The Student's t test was used to calculate p values.

## Results

### Multiple NS5A mutant alleles alone can confer resistance to CsA

**Figure 1:**
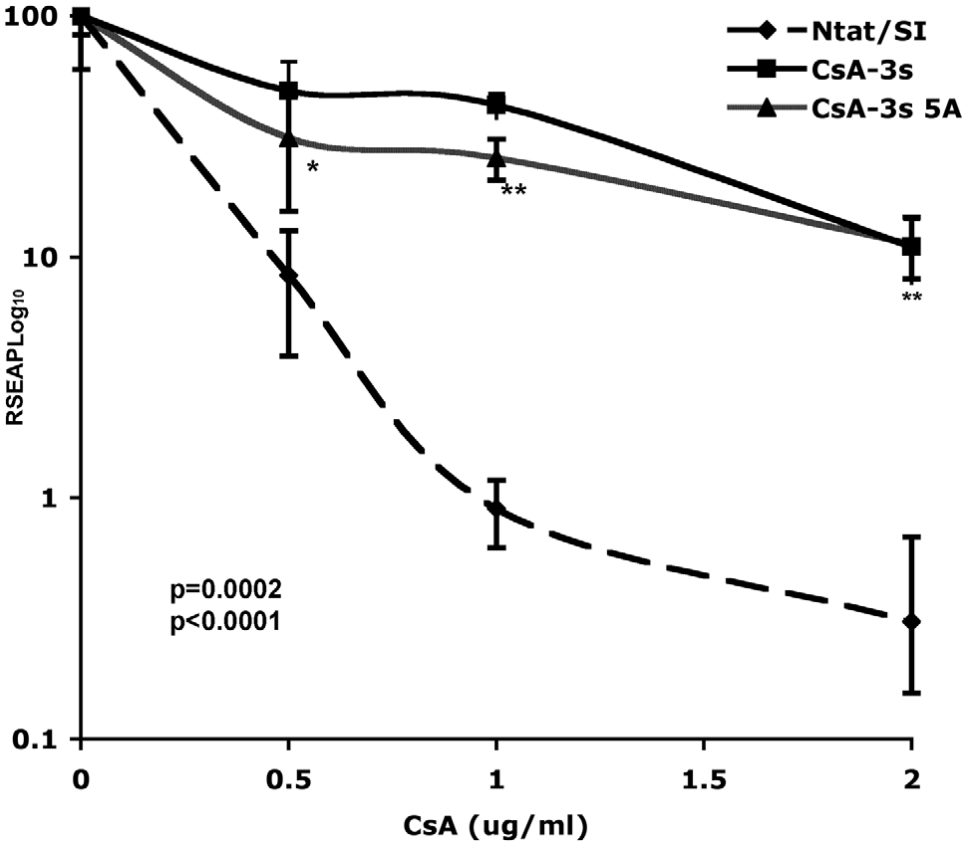
NS5A confers CsA resistance. Equal numbers of replicon cells were seeded for the Ntat/SI, CsA-3s and CsA-3s 5A cell lines. The cultures were treated with CsA at the indicated concentrations for 6 days. Media was changed on day 6 and CsA was added to the cells again. On day 7, media was collected and assayed for SEAP activity. They axis shows RSEAP Log10 (relative SEAP Log values). Student's t test was used to calculate p values. Ntat/SI vs CsA-3s 5A at 1.0 and 2.0 µg/ml <0.0001; at 0.5 µg/ml is 0.0002.

In previously published work [15], we exposed the HCV replicon to CsA for three weeks in culture and selected for relatively resistant mutants. We sequenced replicon RNA from surviving cells and found mutations in both NS5A and NS5B regions. These NS5A and NS5B mutations were introduced into the parental replicon [15]. The resulting replicons have lower susceptibility to CsA compared to wild-type. The mutant replicon CsA-1s 5A with mutations in NS5A alone has identical CsA susceptibility to that seen for the CsA-1s replicon having both NS5A and NS5B mutations [15]. It was unclear if the other NS5A mutants are also able to replicate without mutations in NS5B. All of the mutant replicons share a common set of 5 mutations in NS5A, but differ at 1–2 additional amino acid residues. The CsA-3s replicon is the most resistant of the CsA mutants. The mutant NS5A from CsA-3s has an additional 325 mutation compared to CsA-1s. It also lacks a mutation at 288 present in CsA-1s. The NS5A gene of the CsA-3s replicon was cloned into the background of the parental Ntat/SI replicon and stable replicon cell lines were obtained with G418 selection. This replicon was referred to as CsA-3s 5A. Stable cell lines were obtained for the Ntat/SI, CsA-3s and CsA-3s 5A replicons. All 3 replicons were PCR amplified and sequenced after G418 selection to look for any changes (reversions or additions) that may have arisen during selection. No changes were observed in NS5A in any of the replicons. We then compared the CsA susceptibility of Ntat/SI, CsA-3s and CsA-3s 5A replicons. The absolute SEAP values (a marker for HCV replication) in absence of drug are similar between Ntat/SI and CsA-3s and slightly lower for CsA-3s 5A (Fig. S1). Remarkably, the CsA-3s 5A is equally resistant to CsA as the parent CsA-3s containing both NS5A and NS5B mutations (Fig. 1). The CsA-3s 5A behaves similarly to the CsA-1s NS5A in conferring most of the resistance to the mutant. Thus the role of NS5A in CsA susceptibility is common among different mutants.

### NS5A amino acid residue 321 plays a significant role in resistance

**Figure 2:**
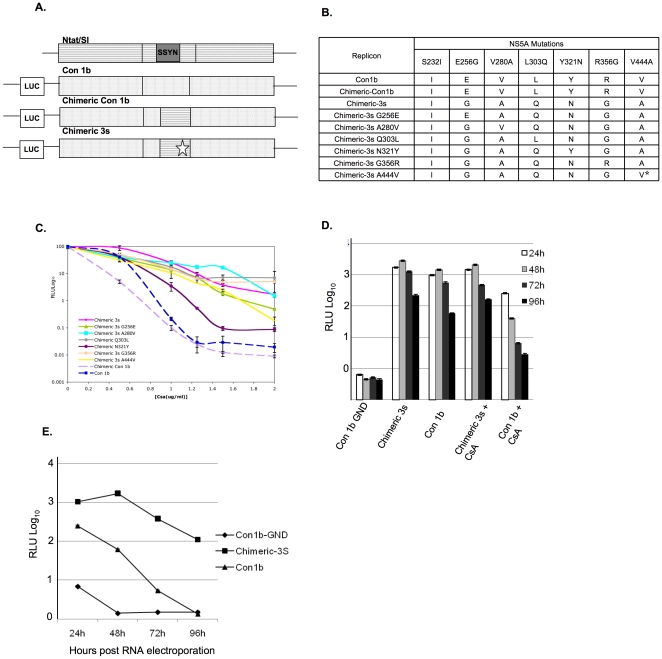
CsA sensitivity of different chimeric 3s replicons. (A) Schematic representation of different Chimeric replicons derived from Con1b and Ntat/SI. Star represents all 3s mutants (acc. no. EF115302), SSYN insert present only in 1bN replicon.  =  Con1b sequence,  =  1bN sequence. (B) List of cyclosporine resistant mutations cloned in a con 1b background. Star indicates reversion of that particular amino acid. (C) Equal numbers of replicon cells were seeded for the Con 1b, Chimeric Con1b, and Chimeric 3s variants. The cells were treated with different concentrations of CsA as indicated on the x axis and assayed for luciferase activity. The y axis shows relative light units Log values (RLULog_10_). Error bars represent standard deviations from three separate experiments read in triplicate. Student's t test was used to calculate p values. Con 1b vs Chimeric 3s N321Y P<0.0001 at 1, 1.5, and 2.0 µg/ml CsA. Chimeric 3s N321Y vs Chimeric 3s P<0.0001 at 1, 1.5, and 2.0 µg/ml CsA. (D) Huh7.5 cells were electroporated with Chimeric Con1b and Chimeric 3s replicon RNA and plated in either the presence or absence of 1.0 µg/ml CsA. Equal numbers of cells were harvested at 24, 48, 72, and 96 hours and analyzed for luciferase activity (RLULog_10_). Three independent RNA electroporations were performed and average luciferase readings are presented for the indicated time points. Error bars represent standard deviation (E) Huh7.5 cells were electroporated as in (D) except the cells were pretreated with 1.0 µg/ml CsA for 6 days and plated in the presence of drug. Equal numbers of cells were collected and luciferase activity was analyzed (RLULog_10_). The average of three independent RNA electroporations is presented. Error bars represent standard deviation.

The CsA-3s contains six point mutations in NS5A. It is unclear if a single mutation accounts for resistance or if the mutations act synergistically with each other. Despite the heterogeneity in NS5A sequence none of these mutations are commonly present in the extensive sequence banks of HCV isolated from patients (see Table 1). We replaced individual NS5A mutations with the wild-type original residue in context of the remaining five NS5A mutations. We chose to revert individual mutations to the wild-type amino acid because individual mutations may not have been viable or functional for altered CsA resistance by themselves. These mutant fragments were amplified without the SSYN 4 amino acid insertion unique to the 1bN strain and cloned into the Con 1b luciferase replicon which allows evaluation of the effect of each mutant in a more typical HCV NS5A structure (Fig. 2A). A list of replicons and mutations are shown in Fig. 2B. Without the SSYN, the amino acid positions of the mutations now change by 4 amino acids with respect to the start of NS5A. A control Chimeric Con 1b was also constructed with the original 1bN NS5A, but lacking the 4 amino acid insertion. Stable cell lines were obtained for the chimeric and wild-type Con 1b replicons. All of the replicons were PCR amplified and sequenced again to confirm the sequence and check for additional mutations. All the replicons retained mutations, and no additional coding compensatory mutations were found except for Chimeric 3s A444V which reverted back to Alanine. The different replicons were treated with various concentrations of CsA for 7 days. The cells were harvested and assayed for luciferase activity. The absolute data is presented in Fig. S2 showing that the luciferase activities in the absence of CsA are similar with the exception of Chimeric 3s A444V which replicates better than the others and after transfection reverted to A such that its coding sequence is identical to Chimeric 3s, but lacks the SSYN insertion. The Chimeric 3s replicon with all 6 CsA resistance mutations but lacking the SSYN insert is still resistant to CsA (Fig. 2C). The Chimeric 3s G260E, Chimeric 3s A280V, Chimeric 3s Q307L and Chimeric 3s A444V mutants show as much resistance as the Con 1bN 3s (Fig. 2C). However, the Chimeric 3s N321Y mutant differs in CsA sensitivity compared to Chimeric 3s. This mutant differs from the Chimeric 3s in having the wild-type tyrosine instead of an asparagine residue at amino acid 321. Its phenotype is intermediate between the Con 1b (p≤0.0001) and Chimeric-3s replicons (p<0.0001). Thus, the tyrosine at residue 321 in NS5A appears to play an important role in CsA sensitivity.

**Table 1:** Conservation of amino acids at positions identified by 3s mutant in HCV sequenced from genotype 1 patients.

Amino Acids	Genotypes					Comments
	1b	1a	1	1g	1c	
Gly 260	178/1203	43/208	115/115	1/1	1/2	High frequency of D,A,Q at position 260 for 1b,High frequency of A,T,Q,M at position 260 for 1a
Val 284	140/1203	38/208	45/115	0/1	1/2	All non V positions were Ile
Leu 307	1200/1203	208/208	115/115	1/1	2/2	2 L to P changes AM401923, DQ492170,1 L to Q change DQ491971
Asp 320	1196/1203	208/208	115/115	0/1	1/2	3 D to E changes AF033364,AY808017, EF4075003D to G changes AM493473, DQ492132, DQ492178 1D to N change AY8080331G = AM910652
Tyr 325	1197/1203	208/208	115/115	1/1	2/2	2 Y to C changes AM401892, AM4019742 Y to D changes DQ491959, DQ4919651 Y to H change AF0333611 Y to N change DQ492001
Val 356	1198/1203	208/208	115/115	1/1	2/2	2 R to Q change1 R to W change2 R to L change
Gly 448	1097/1203	208/208	115/115	1/1	2/2	4 V to I changes AF033364, AY808017,EF407500 1 V to A change AU4022251 V to G change AM402246

Transient replication assays were also performed to determine the significance of NS5A mutations for CsA susceptibility. Replicon RNA was electroporated into cells that were untreated or treated with CsA and harvested at different time intervals to examine the luciferase activity. The Chimeric 3s and Con1b replicon replicate to similar levels (Fig. 2D). When CsA was added to the Con1b and Chimeric 3s following RNA electroporation, the Chimeric 3s replicates more effectively than wild- type (Fig. 2D). This effect was even more pronounced when cells were pretreated with CsA prior to RNA electroporation (Fig. 2E). As shown in Fig. 2E, 72 hours after electroporation in the presence of CsA, the Chimeric 3s replicon replicates to ∼100 fold higher levels than the wild-type. The Chimeric 3s N321Y with a single wild-type 321 residue (Y) and the remaining five 3s mutations replicated poorly when compared with the other single revertants in similar transient replication assays (data not shown).

**Figure 3:**
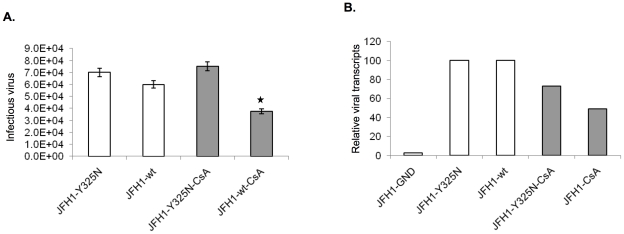
Replication of JFH1 infectious clone carrying a Y325N mutation in the presence and absence of CsA. (A) Infectivity of in vitro-transcribed RNA from JFH1-Y325N and JFH1-wt were determined by infectious center assay. Huh7.5 cells were electroporated with RNA transcripts and cleared supernatant was examined for number of infectious particles by serially diluting and passing on to fresh Huh7.5 cells. The infectious centers were monitored by immunofluorescence assay by using anti-NS5A monoclonal antibodies. Y axis shows number of infectious virus produced. The average of two independent experiments with error bar representing standard deviation is presented. Student's t test was used to calculate p values. JFH1-wt vs JFH1-wt-CsA, p = 0.012, indicated by star. (B) Northern blot analysis of total RNA from either non treated or pretreated Huh7.5 cells electroporated with RNA from JFH1-Y325N and JFH1-wt. Total RNA was isolated from these cells and analyzed by probing with ^32^P-labeled random probe derived from C-terminal portion of NS5A. The signal was quantified with Storm and the reading was normalized with respect to actin control. The y axis represents relative transcript levels with JFH1-Y325N arbitrarily set to 100%.

To further evaluate the role of the 321 mutation by itself, we engineered the 321 mutation in the background of the JFH1 infectious clone. The JFH1-Y325N and JFH1-wt produce a similar number of infectious viral particles in the absence of CsA (Fig. 3A). However, in the presence of CsA (gray bars), JFH1-wt produces about 50% less infectious particles than JFH1-Y325N (Fig. 3A). The viral replication was further examined by northern blot assay and revealed similar levels of replication for both infectious clones in normal Huh7.5 cells. But the JFH1-wt virus displays approximately 20% less replication compared to JFH1-Y325N in presence of CsA, demonstrating the crucial role of residue Y at position 325 in CsA resistance (Fig. 3B).

### Mutant NS5A does not differ from wild-type in binding NS5B

**Figure 4:**
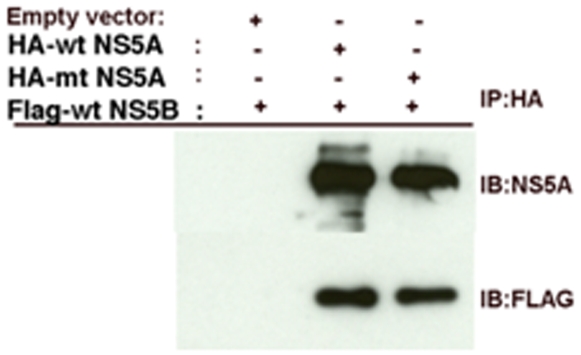
Wild-type and mutant NS5A's do not differ in binding NS5B. 293T cells were co-transfected with either HA-wt NS5A or HA-mt NS5A together with FLAG-wt NS5B (Con 1b). The cells were harvested 48 hours after transfection and immunoprecipitated with HA-specific antibody. Immunoprecipitates were western blotted and probed with monoclonal antibody specific for NS5A and anti-FLAG antibody to detect NS5A and NS5B respectively. Signal was detected by chemiluminescence assay (IP-immunoprecipitation, IB-immunoblot).

NS5A binding to NS5B maps to two regions of NS5A, amino acids 105–162 and 277–334 [16]. As the 321 residue of NS5A lies in the second NS5B interaction site, it is possible that the mutant NS5A differs from wild-type in NS5B binding. To investigate the NS5A-NS5B interaction, 293T cells were transfected with either HA-wt NS5A (1bN, wild-type) or HA-mt NS5A (CsA-3s, mutant), along with FLAG-tagged NS5B. Cell lysates were immunoprecipitated with HA-specific antibody and probed with anti-FLAG mAb to detect NS5B. We detected no difference in binding NS5B between wt NS5A and mt NS5A (Fig. 4, lanes 2 and 3). The pull down of NS5B is specific since no NS5B is brought down with the empty vector control (Fig. 4, lane 1).

### Both CypB and CypA directly bind NS5A, and this binding requires cyclophilin catalytic residues

**Figure 5:**
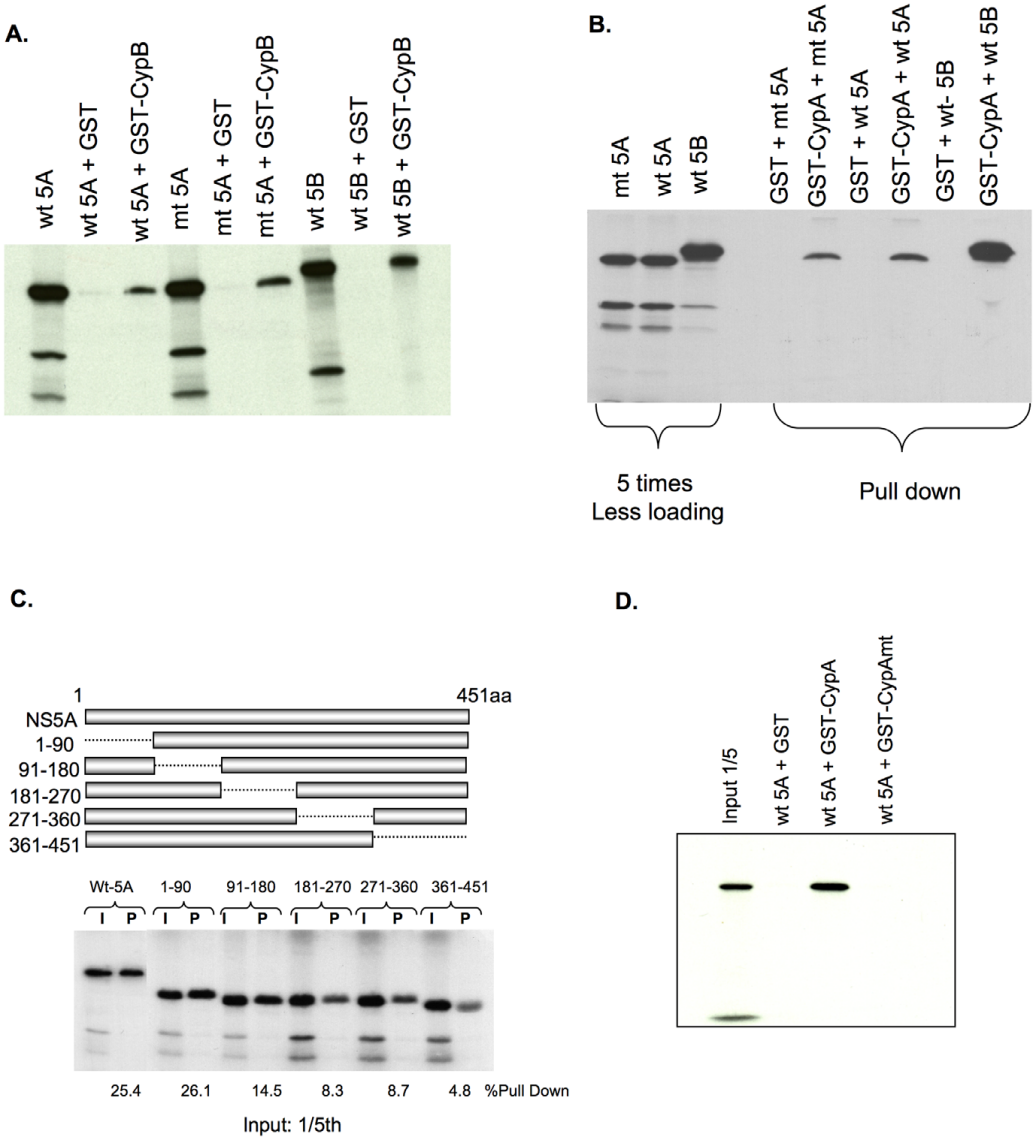
NS5A binds both CypB and CypA. (A) wt-NS5A, mt-NS5A, and wt NS5B-FLAG (1bN) were labeled with ^35^S methionine/cysteine by in vitro translation. Labeled proteins were incubated with either GST alone (Lanes 2, 5, and 8) or GST-CypB (Lanes 3, 6, and 9). Gluthathione Sepharose beads were used to purify GST-containing complexes from the binding mixture. After separation via SDS-PAGE, ^35^S-labeled proteins were detected by autoradiography. Lanes 1, 4, and 7 contain labeled proteins used in the binding assay. (B) The wt-NS5A, mt-NS5A, and wt NS5B-FLAG (Con 1b) were in vitro translated and incubated with either GST alone or GST-CypA. The GST binding assay was performed as described in (A). The left three lanes contain labeled proteins used in the binding assay. (C) The binding region within the NS5A region for CypA was mapped by generating 90 amino acids in-frame deletion mutants. The radiolabeled mutant proteins were incubated with GST-CypA as above. Input protein (I) was loaded at 1/5 volume alongside the corresponding pull-down product (P). Pulled down signal was quantified using Storm and % values of pull down are shown for each deletion mutant. (D) The CypA containing mutations in the isomerase active site was analyzed for binding to wt-NS5A using GST binding assay.

It has previously been shown that NS5B binds both CypB and CypA [13], [14]. However, prior genetic evidence suggested that NS5A, not NS5B, has the largest effect on CsA resistance [15]. Previous studies did not address the role of NS5A in cyclophilin binding. To determine if CypB interacts with NS5A, HA-wt NS5A and HA-mt NS5A were in vitro translated with ^35^S methionine/cysteine and proteins were incubated with GST-CypB or GST alone. The in vitro translated NS5B was used as a positive control for CypB binding. Both wt NS5A and mt NS5A interact with CypB (Fig. 5A). GST alone did not show an interaction with either version of NS5A. We also tested if NS5A bound CypA, and found a more pronounced difference between the binding of HCV proteins to CypA compared with CypB (Fig. 5B). This data suggests that for CypB, NS5A binds CypB almost as well as NS5B does, whereas for CypA, the CypA:NS5A interaction is weaker than the CypB:NS5B interaction. We also tested whether or not recombinant NS5A purified from E. coli binds GST only when fused to CypA and if unrelated proteins such as CFP bind GSTCypA. The CFP along with other Ni^++^ purified proteins are shown in supplemental data NS5A (Fig. S5B) confirming that the GSTCypA fusion protein does not bind all proteins with prolines present and that the GSTCypA:NS5A interaction is specific.

All of the CsA resistant mutations fall in domain 2 and 3 of NS5A. To determine which region of NS5A was critical in binding CypB, N terminal deletions of wild-type and mutant CsA-3s NS5A were constructed which lacked domain 1 and binding was performed as above. Both wt D23 NS5A and mt D23 NS5A bind CypB with same efficiency (supplemental data, Fig. S3). Thus, domains 2 and 3 of NS5A are sufficient to bind both CypB and CypA. We mapped the NS5A interacting region with CypA by generating NS5A mutants containing 90 amino acid in-frame deletions and performed a CypA binding assay. We observed much of the binding maps to the C-terminal region of NS5A, in particular amino acids ranging from 361–451 where a majority of the binding interaction is lost and only 5% of input is retained (Fig. 5C). The NS5A deletion ranging from amino acids 271–360 binds approximately 8–9% of the input NS5A, while deletion of the first 180 amino acids does not have a major defect in binding to NS5A. This data indicates the CypA binding regions are clustered in second half of NS5A in between amino acids 181–451.

The Prolyl-peptidyl Isomerase (PPI) activity of CypA catalyzes the cis-trans isomerization of peptide bonds that connect prolines. To examine the role of the catalytic domain of CypA in HCV NS5A binding we converted the two amino acids (R55 and F60) in the catalytic domain to alanine. The mutant protein was then tested for its ability to pull down NS5A. Using the GST pull down assay, the CypA mutant protein displays complete loss of binding to NS5A indicating crucial involvement of the active site residues in NS5A binding (Fig. 5D).

### Mutant NS5A facilitates the interaction of NS5B and CypB in cell culture

**Figure 6:**
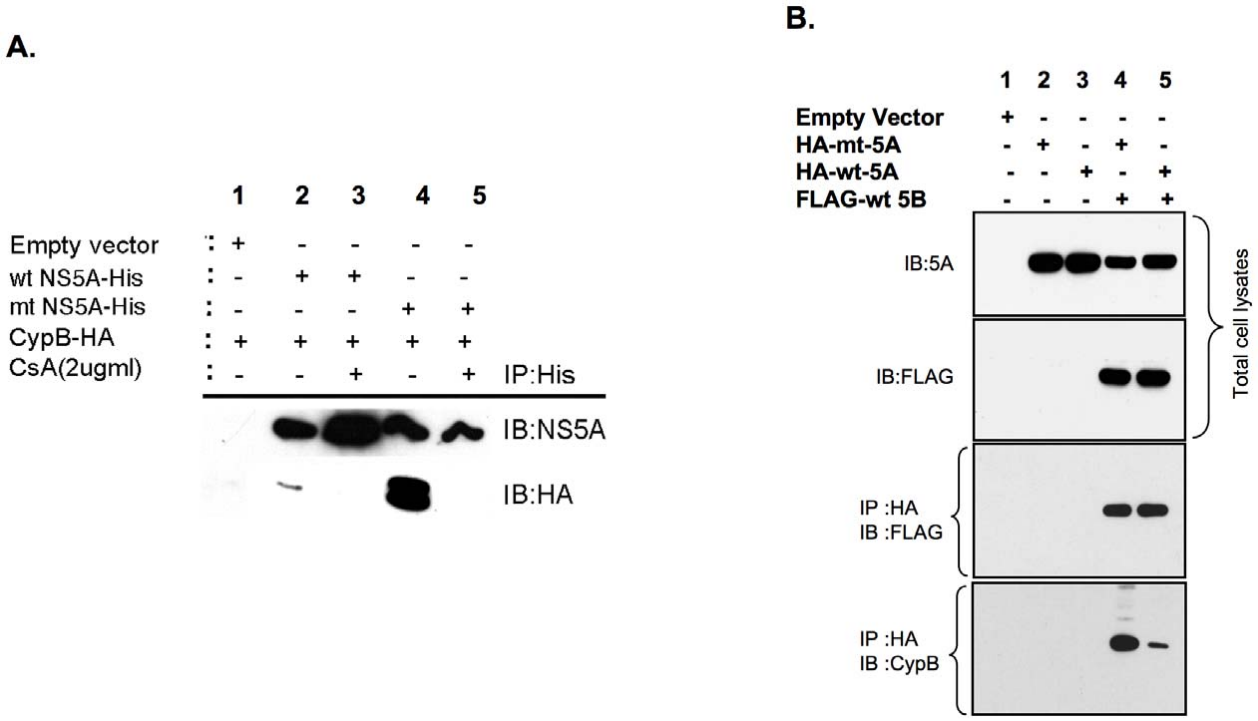
Mutant NS5A binds CypB better than wild-type NS5A in cells. (A) 293T cells were co-transfected with either wt-NS5A-His (lanes 2 and 3) or mt- NS5A-His (lanes 4 and 5) together with HA-tagged CypB in the presence and absence of 2.0 µg/ml CsA. Forty eight hours after transfections, cell lysates were immunoprecipitated with anti-His antibody. The immunoprecipitates (IP) were Western blotted and probed with NS5A-specific and HA-specific antibodies to detect NS5A and CypB respectively. Lane 1 represents transfections with an empty vector control. (B) 293T cells were co-transfected with either HA-wt NS5A (lanes 3 and 5) or HA-mt NS5A (lanes 2 and 4) in the presence (lanes 4 and 5) or absence (lanes 1–3) of FLAG-wt NS5B (Con 1b). Lane 1 represents transfections with an empty vector control. Forty eight hours after transfections, cell lysates were immunoprecipitated with anti-HA antibody and Western blotted. The blots were probed with antibodies specific for NS5A (top blot), FLAG (to detect NS5B (middle blot), and CypB (lower blot) respectively. Upper two blots show total cell lysate without HA-specific immunoprecipitation.

To determine if the interaction between NS5A and CypB occurs in vivo, 293T cells were co-transfected with His-tagged wt-NS5A or mt- NS5A along with CypB-HA. The cells were lysed and immunoprecipitated with anti-His antibody to purify NS5A-containing complexes. Western blots were probed with both NS5A-specific antibodies and HA-specific antibody to detect CypB. Both wt NS5A and mt NS5A interact with CypB-HA (Fig. 6A, lanes 2 and 4). However, mt NS5A binds substantially greater amounts of CypB than wt NS5A. CypB-HA is not immunoprecipitated in the absence of NS5A (Fig. 6A, lane 1, empty vector control). Co-transfections of wild-type and mutant NS5A with CypB are both disrupted by treatment of transfected cells with 2 µg/ml of CsA (Fig. 6A, lanes 3 and 5). Several attempts were made to pull down endogenous CypB from cells using transfected and replicon cells, however no CypB can be pulled down by NS5A alone (data not shown). However, transfected NS5A is successful at pulling down endogenous CypB (similar to replicon setting) only in the presence of NS5B (Fig. 6B, lanes 4 and 5). It is possible that when CypB is present in large amounts, it can out compete other cellular factors that target NS5A. However in the presence of replicon levels of HCV proteins and endogenous CypB, the lower levels of CypB do not stably out compete the other cellular NS5A binders unless NS5B is present to facilitate NS5A interaction with CypB. Nevertheless, the mutant's interaction with NS5B is enhanced beyond that of wild-type NS5A. Similar coimmunoprecipitations were performed in Huh 7.5 cells and showed the same results (Fig. S4).

## Discussion

Cyclophilins have now been implicated in the life cycle of several different DNA and RNA viruses [25], [26], [27], [28]. In one particularly well-studied example, CypA binds the HIV Gag protein and is incorporated into the virion in addition to binding incoming HIV capsid protein. However, the mechanistic role of CypA in HIV remains unclear despite intense investigation and detailed structural insights [29], [30]. It was initially proposed that CsA inhibits HCV by blocking the interaction between the active site of CypB and the viral polymerase NS5B [6]. Although NS5B has largely been thought of as the principal mediator of CsA action, genetic mapping of CsA resistance implicated NS5A as being the principal gene [15] and recently it was shown that the presence of NS2 also modulates CsA susceptibility [31] although in the case of NS2 it remains unclear if NS2 directly bind cyclophilins. The main finding in the current study is that NS5A directly binds cyclophilins, and that its interaction is weaker in vitro than that of NS5B and cyclophilins. Since cyclophilin: HCV interactions also occur in vivo [10], our data suggests NS5A variants may have the largest effect on susceptibility and clinical resistance to cyclophilin inhibitors will arise first in NS5A mutants before NS5B mutants. We previously selected HCV replicons that could grow in the presence of CsA [15]. Mutations mapped to both NS5A and NS5B regions. Several groups have also found that NS5A mutations alter the sensitivity of the replicon to cyclophilin inhibitors in cell culture [32], [33]. We show a cell free direct interaction with both CypA and CypB, recent evidence support that CypA not CypB is the most relevant cyclophilin in most Huh7 cell lines [13], although nonCypA cyclophilins may play a role in some cell lines [33].

The barrier to resistance for CsA is relatively high compared to other antivirals in which a single mutation confers substantial resistance. The different mutant NS5A sequences localize to domains 2 and 3. Here, we provide data that shows mutations from one strain (1bN) also affects other genotype 1b strains (Con1b) and even a genotype 2a (JFH) (Fig. 3). The affect on JFH is despite the previously reported observation that JFH replication is not as dramatically dependant on cyclophilins [11]. HCV is classified into 6 divergent genotypes and has extensive sequence variation even within each genotype. None of these CsA resistance mutants occur frequently in any of the 6 genotypes. Due to the large amount of error prone replication that occurs in patients over decades, even variants with some fitness cost may occasionally become fixed in an individual. We examined >1200 genotype 1 sequences and found that our in vitro selected mutations did not occur frequently. Remarkably little variation occurred at 321, and the Y to N variant at 321 is present in less than 0.1% of genotype 1 (Table 1). While some of these sequences were likely from HCV infected transplant patients, the clinical data associated with these sequences is insufficient to determine with certainty if any specific patient was exposed to CsA.

A Chimeric 3s N321Y replicon was constructed restoring the asparagine mutation at amino acid position 321 to the wild-type tyrosine residue. This mutant replicon, contains all of the other CsA-3s mutations, but lacks the mutation at 321. Chimeric 3s N321Y is more sensitive to CsA treatment than Chimeric 3s suggesting a tyrosine residue at position 321 contributes to some but not all of the CsA sensitivity. This mutation is in a region of NS5A that binds NS5B [16] and targets a residue previously shown to be critical for RNA replication [34]. However, our studies showed that mutant does not differ from wild-type in its interaction with NS5B (Fig. 4). Even the most structured part of NS5A, domain I, apparently has multiple conformations [35], [36]. Therefore, it is hard to know the conformational effect of the tyrosine at residue 321 NS5A in domain 2 at this time. Certainly a conformational effect could explain the observed difference between the Con1b and Chimeric-3s replicons in cyclophilin binding. The interaction of truncated NS5A molecules with cyclophilins localizes the CypA: NS5A and CypB: NS5A binding region(s) to domains 2 and 3 of NS5A. The NS5A residues 360–440 provide slightly more of the binding affinity than 270–360. The importance of the 321 residue that we show here is consistent with a recent NMR structural study provided evidence that for at least JFH2a NS5A, domain 2, including residue 321 specifically makes an important interaction with cyclophilins (particularly CypA which binds a larger motif than CypB). Still peptides containing 321 accounted for only one tenth of the binding strength of all domain 2 [37]. It is possible that 321 variation results in a conformational change in NS5A that may affect other residues either adjacent or more distant. Our data suggests that at least for genotype 1 NS5A, a domain 3 cyclophilin interaction may also be important.

The interaction between NS5B and cyclophilins has been demonstrated by several groups. In one study, CsA was used to select mutations in NS5B using a HCV replicon with GFP inserted into NS5A [38]. The GFP-containing NS5A may be distinct in conformation from wild-type. It is possible that NS5B: Cyp interaction is the most CsA susceptible genetic locus when GFP is inserted in domain 3 of NS5A, while the NS5A: Cyp interaction is the more important locus in a non-tagged NS5A.

Previous functional studies have investigated the interaction of NS5B alone and cyclophilins. Here, we show a novel interaction between NS5A and both CypA and CypB. Mutant NS5A binds CypB to a greater extent than wild-type in cells (Fig. 6 and S4). In this study with tagged CypB the wild-type interaction was barely detected while the mutant interaction was strong (Fig. 6A). The GST pull-down assay for CypA binding does not reflect a difference between wild-type and mutant NS5A in either CypA or CypB (Fig. 5). We speculate that this discrepancy is due to the absence of other NS5A cellular binding partners in the GST assay.

We made several attempts to immunoprecipitated endogenous CypA and CypB with NS5A (data not shown). Neither endogenous CypA nor CypB could be pulled down by NS5A expressed alone in cells. It is possible the NS5A: cyclophilin interaction cannot stably compete with other NS5A partners in the absence of other viral proteins such as NS5B. NS5A could pull down endogenous CypB only in the presence of NS5B (Fig. 6B). NS5A did not immunoprecipitate CypA even in the presence of NS5B (data not shown). Endogenous CypB levels in Fig. 6B may primarily reflect NS5B pull down of CypB. However, such a possibility is unlikely as the amount of CypB immunoprecipitated would have been relatively equal between wild-type and mutant NS5A. In either case, mutant NS5A either by itself or through its interaction with NS5B has an enhanced CypB interaction.

It is unclear if cyclophilins assist in folding NS5A and NS5B or, alternatively, form part of the HCV replication complex. It is possible that cyclophilins allow both NS5A and NS5B to access important conformations. Alternately, binding of both NS5A and NS5B to cyclophilins may facilitate incorporation of cyclophilins into the replication complex. Either way the catalytic portion of cyclophilins is critically involved in NS5A (Fig. 5D) just as it is in NS5B [8]. We cannot genetically dissect if cyclophilins only need to bind HCV proteins, or if they bind and catalyze isomerization around the proline bond. CsA disrupts NS5A's interaction with both CypA and CypB (Fig. 6A) as well as NS5B and CypB (data not shown). While the downstream effects of this disruption are unclear, an enhanced interaction of 3s mutant NS5A to both CypA and CypB could allow the mutant to retain either cyclophilin at low concentrations of CsA. Further study into the details of NS5A interactions with cyclophilins should shed light on how best to pair non immunosuppressive cyclophilin inhibitors with other small molecules to inhibit viral proteins in the replicase complex.

## Supporting Information

Figure S1 The absolute value of data presented in Fig. 1. (ASEAP Log10 indicates the absolute SEAP Log values)
(0.15 MB TIF)

Figure S2 The absolute value of data presented in Fig. 2B. (ALUL Log10 indicates absolute light unit Log values)
(0.25 MB TIF)

Figure S3 NS5A constructs without domain1 (wt D23 NS5A, mt D23 NS5A) were translated in vitro and incubated with either GST alone or GST-CypB as described in Fig. 5A. 35S labeled GFP protein was used as a negative control for binding.
(0.80 MB TIF)

Figure S4 Mutant NS5A binds CypB better than wild-type NS5A. Huh7.5 cells were co-transfected with either HA-wt NS5A or HA-mt and wt NS5B (Con 1b) in the presence of CypB-FLAG tagged plasmid. Forty eight hours after transfections, cell lysates were immunoprecipitated with anti-HA antibody and Western blotted with anti-FLAG (bottom panel). The top panel shows probing the total cell extract with anti-FLAG antibodies to demonstrate equivalent CypB expression.
(0.38 MB TIF)

Figure S5 HCV NS5A binds CypA A) Coomassie staining of HIS-tagged E. coli purified proteins B) Western blot analysis using anti-HIS monoclonal antibody demonstrates NS5A in the GSTCypA complex but not in GST, and no CFP binds either GST or GSTCypA.
(3.00 MB TIF)
