# Supplementary figures and images for: Cyclosporine Inhibits a Direct Interaction between Cyclophilins and Hepatitis C NS5A
Source: PLoS One. 2010 Mar 23;5(3):e9815. doi: 10.1371/journal.pone.0009815 (PMC2843741; doi:10.1371/journal.pone.0009815)

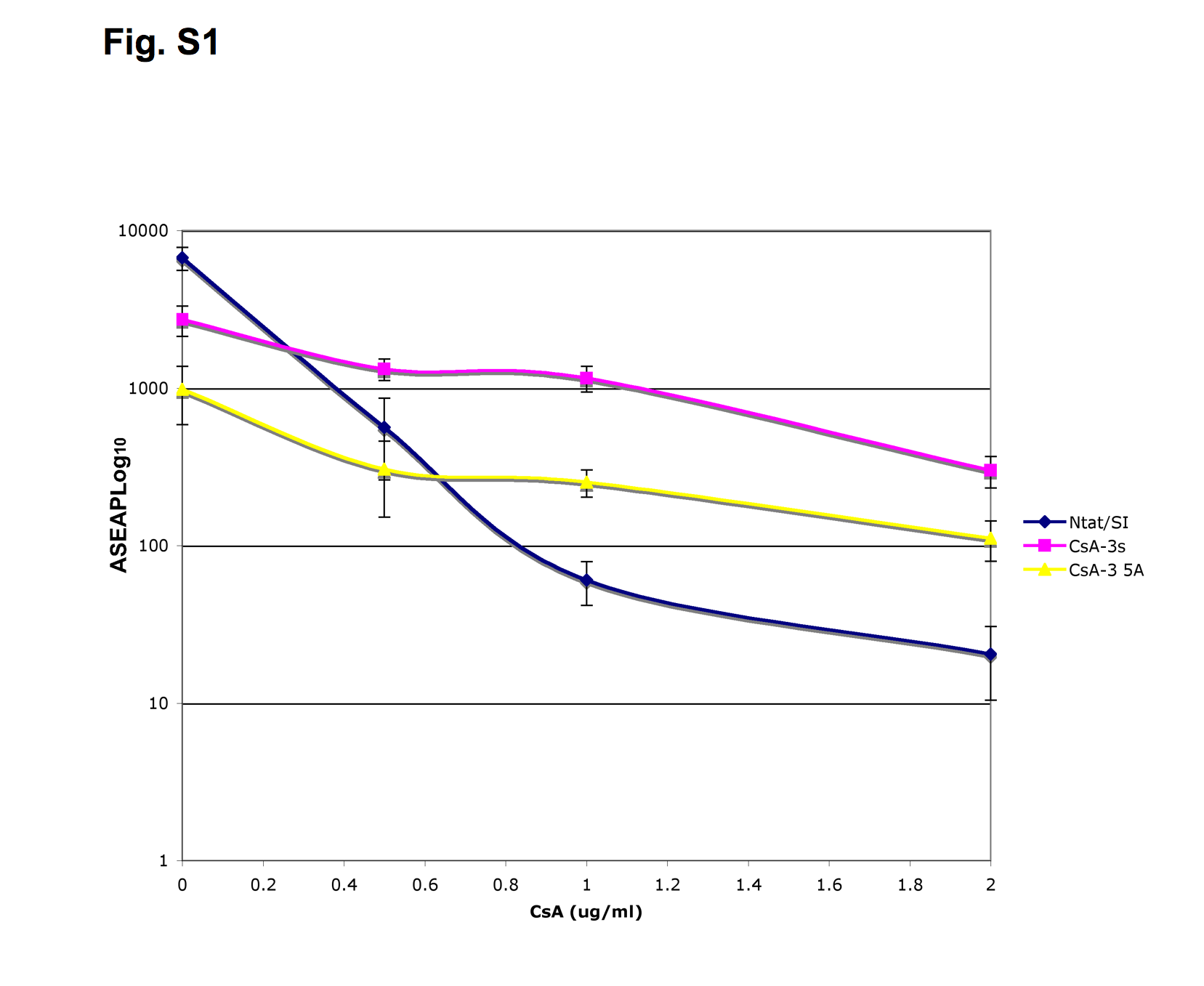

Supplement: Figure S1 — The absolute value of data presented in Fig. 1. (ASEAP Log10 indicates the absolute SEAP Log values) (0.15 MB TIF) [file pone.0009815.s001.tif]

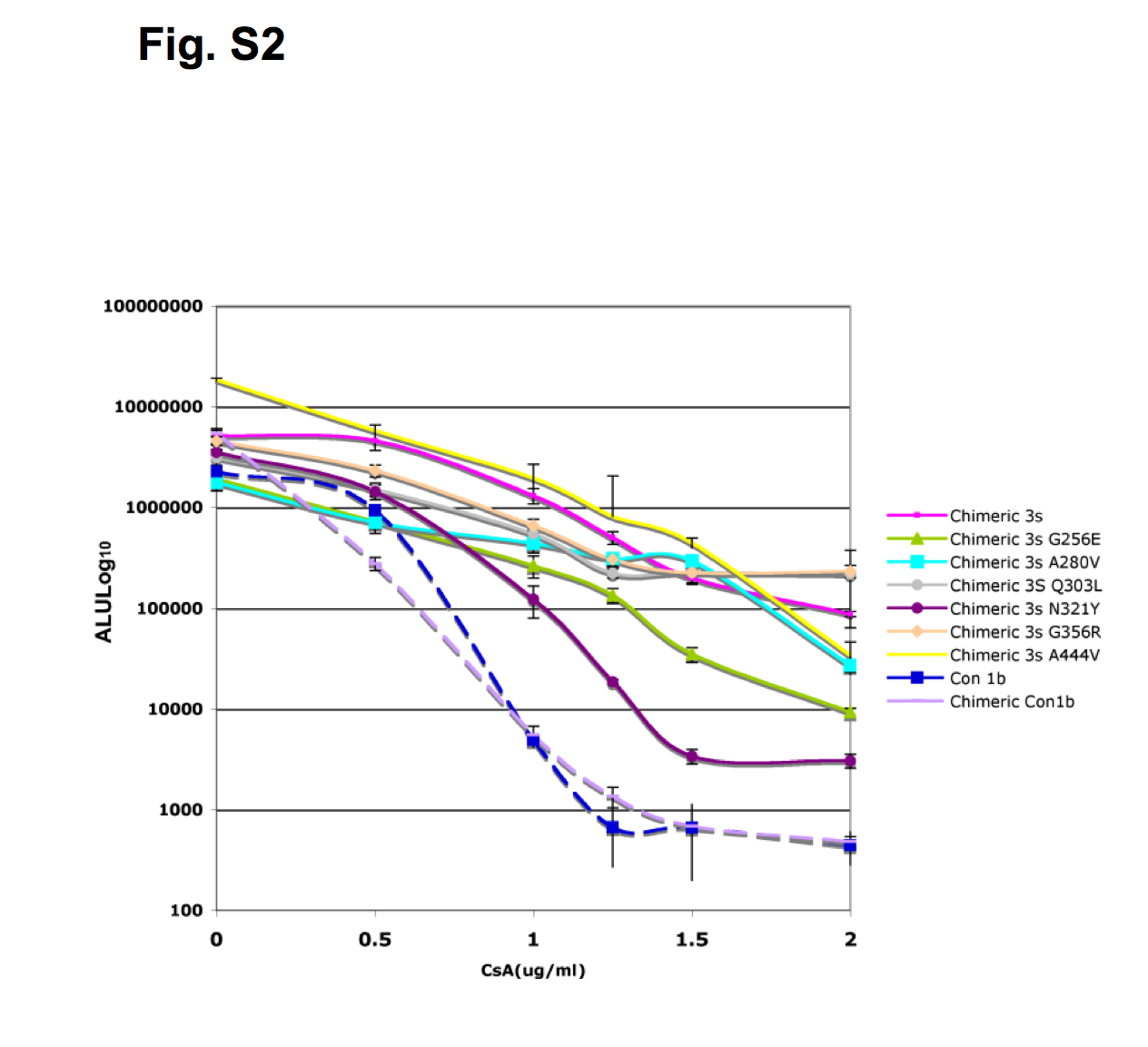

Supplement: Figure S2 — The absolute value of data presented in Fig. 2B. (ALUL Log10 indicates absolute light unit Log values) (0.25 MB TIF) [file pone.0009815.s002.tif]

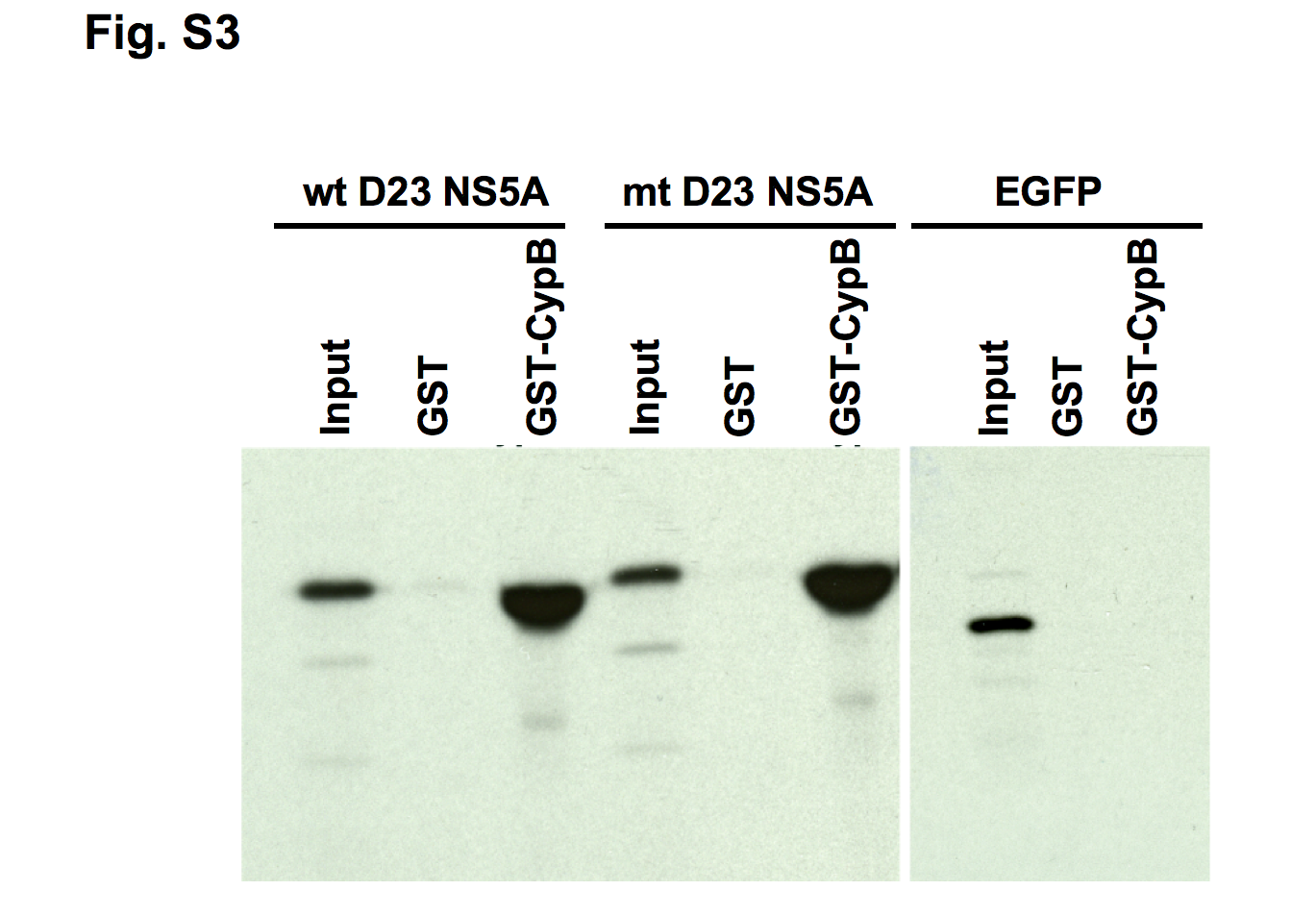

Supplement: Figure S3 — NS5A constructs without domain1 (wt D23 NS5A, mt D23 NS5A) were translated in vitro and incubated with either GST alone or GST-CypB as described in Fig. 5A. 35S labeled GFP protein was used as a negative control for binding. (0.80 MB TIF) [file pone.0009815.s003.tif]

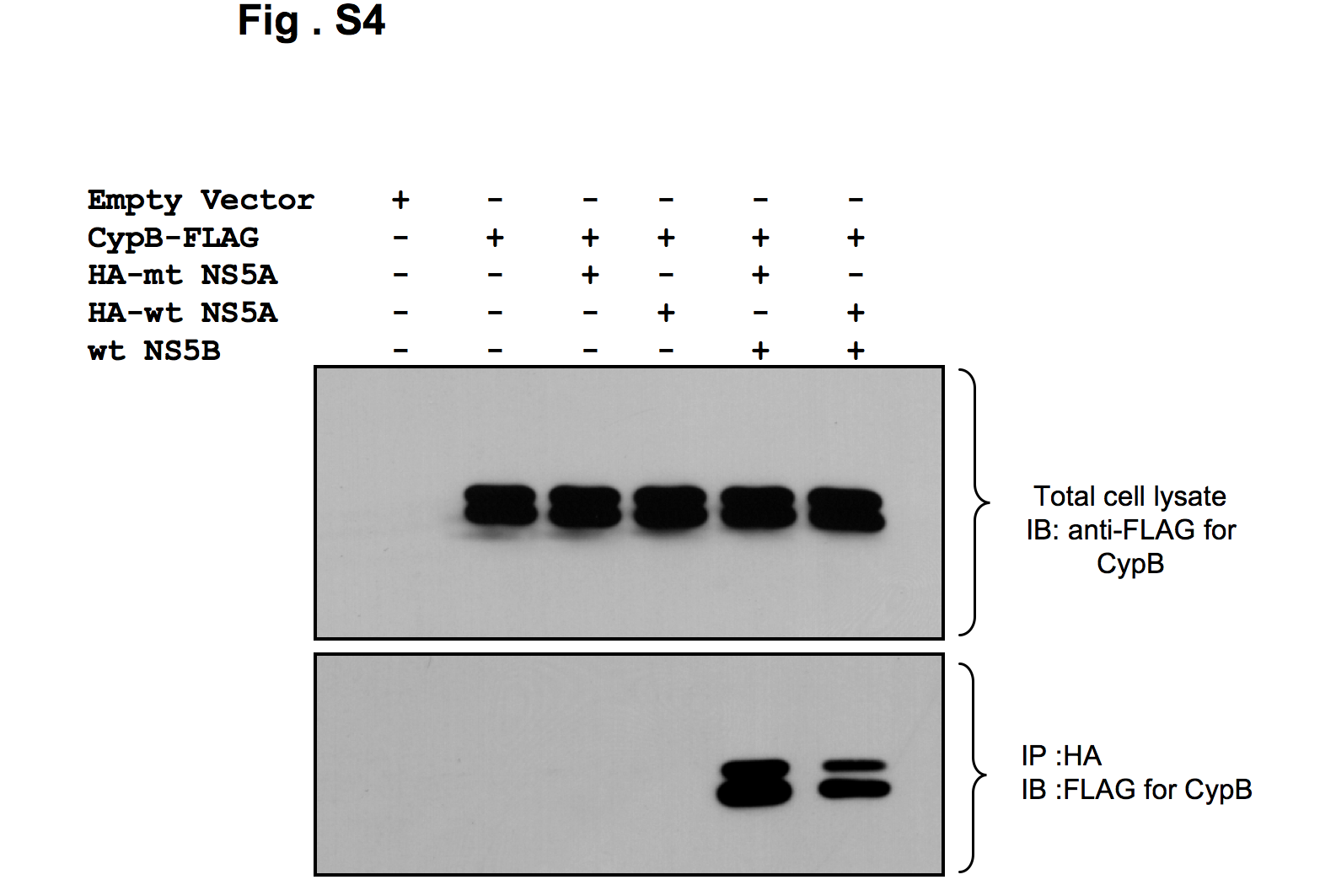

Supplement: Figure S4 — Mutant NS5A binds CypB better than wild-type NS5A. Huh7.5 cells were co-transfected with either HA-wt NS5A or HA-mt and wt NS5B (Con 1b) in the presence of CypB-FLAG tagged plasmid. Forty eight hours after transfections, cell lysates were immunoprecipitated with anti-HA antibody and Western blotted with anti-FLAG (bottom panel). The top panel shows probing the total cell extract with anti-FLAG antibodies to demonstrate equivalent CypB expression. (0.38 MB TIF) [file pone.0009815.s004.tif]

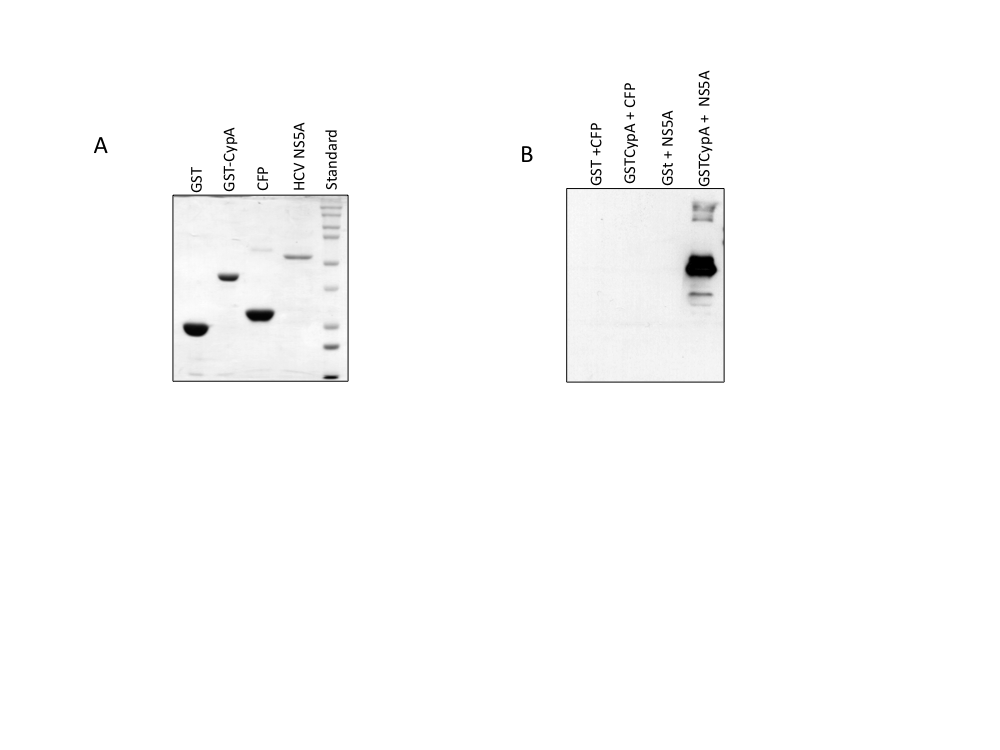

Supplement: Figure S5 — HCV NS5A binds CypA A) Coomassie staining of HIS-tagged E. coli purified proteins B) Western blot analysis using anti-HIS monoclonal antibody demonstrates NS5A in the GSTCypA complex but not in GST, and no CFP binds either GST or GSTCypA. (3.00 MB TIF) [file pone.0009815.s005.tif]
